# Author Correction: Two-component anomalous Hall effect in a magnetically doped topological insulator

**DOI:** 10.1038/s41467-021-24218-1

**Published:** 2021-06-25

**Authors:** Nan Liu, Jing Teng, Yongqing Li

**Affiliations:** 1 Beijing National Laboratory for Condensed Matter Physics, Institute of Physics, Chinese Academy of Sciences, Beijing, 100190 China; 2 School of Physical Sciences, University of Chinese Academy of Sciences, Beijing, 100049 China; 3 Beijing Key Laboratory for Nanomaterials and Nanodevices, Beijing, 100190 China

Correction to: *Nature Communications* 10.1038/s41467-018-03684-0, Published Online 29 March 2018.

The original version of this Article omitted the following sentence from the Acknowledgements: “J.T. acknowledges support from the Youth Innovation Promotion Association of Chinese Academy of Sciences”. This sentence has now been appended at the end of Acknowledgements in both the PDF and HTML versions of the Article.

