# Supplementary material for: Two-component anomalous Hall effect in a magnetically doped topological insulator
Source: Nat Commun. 2018 Mar 29;9:1282. doi: 10.1038/s41467-018-03684-0 (PMC5876350; doi:10.1038/s41467-018-03684-0)
Supplement: Supplementary file 1 — Supplementary Information(PDF 995 kb) [file 41467_2018_3684_MOESM1_ESM.pdf]

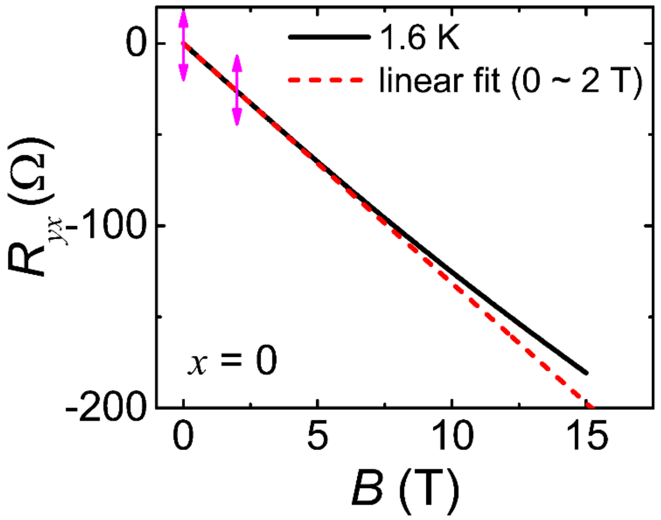

Supplementary Figure 1. Linear range of the Hall effect in a typical Bi_2Se_3 thin film. The Hall resistance data (black solid curve) were recorded from a 10 nm thick Bi_2Se_3 film at T = 1.6 K. The red dashed line is a linear fit of the Hall resistance R_{yx} from B = 0 to 2 T. The deviation from the linear dependence is no longer negligible for the magnetic fields above 5 T due to coexistence of the surface and bulk carriers. Please see Supplementary Note 1 for a detailed discussion of the nonlinear Hall effect in the two-band model.

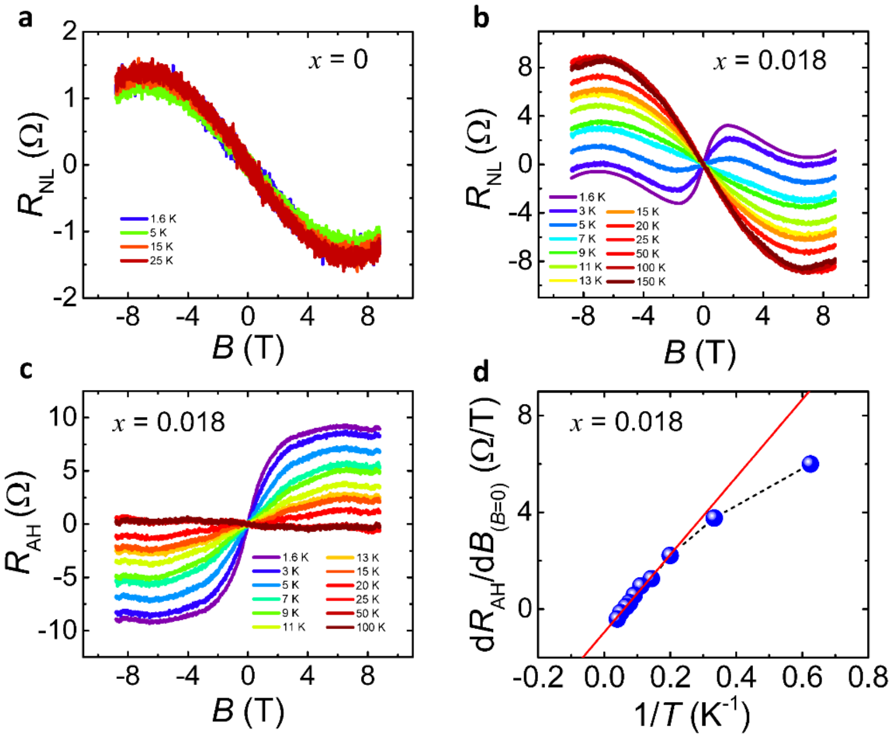

Supplementary Figure 2. Temperature dependences of the nonlinear part of the Hall effect in $(Bi_{1-x}Mn_x)_2Se_3$ thin films. (a,b) Nonlinear part of the Hall resistances of Sample A ($x = 0$) and Sample B ($x = 0.018$). It is defined as $R_{\mathrm{NL}}(B) = R_{yx}(B) - R_{\mathrm{H}}B$, in which Hall coefficient R_{H} is determined by a linear fit of the R_{yx} data at B = 5-9 T. In panel (b), the curves fall onto each other nearly perfectly for $T \geq 25$ K, at which the magnetic order is no longer relevant. (c) The anomalous Hall (AH) resistances of Sample B, obtained by subtracting R_{NL} of nonmagnetic origin from the total signal, i.e. $R_{\mathrm{AH}}(B,T) = R_{\mathrm{NL}}(B,T) - R_{\mathrm{NL}}(B)|_{T=150\,\mathrm{K}}$. When $T \geq 25$ K, R_{AH} nearly vanishes due to a transition to a paramagnetic phase. (d) Temperature dependence of $dR_{\mathrm{AH}}(B)/dB$ at the low field limit for Sample B. The deviation from the linear dependence on $1/T$ suggests a non-paramagnetic order at $T < 5$ K.

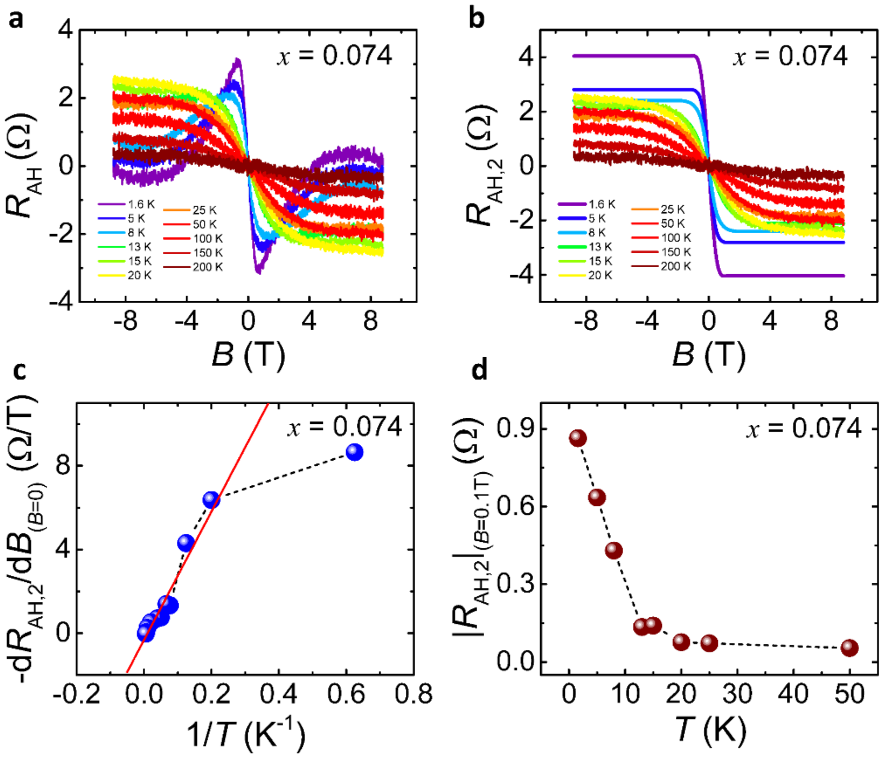

Supplementary Figure 3. Temperature dependence of the anomalous Hall effect in a $(Bi_{1-x}Mn_x)_2Se_3$ thin film with x = 0.074 (Sample F). (a) AH resistances at temperatures from 1.6 K to 200 K. At T < 15 K, the oscillatory AH resistance, $R_{AH,}$ can be separated into two components with opposite signs. (b) The negative R_{AH} components, denoted as $R_{AH,2}$, at various temperatures, which are obtained by following the second method described in the Supplementary Note 2 and Supplementary Figure 6. The negative component persists to higher temperatures than the positive component. The former is related to the surface magnetism, whereas the latter arises from the bulk magnetization. (c) $-dR_{AH}(B)/dB$ at the low field limit plotted as a function of 1/T. At $T <$ 5 K, a deviation from the linear 1/T dependence takes place. (d) Temperature dependence of $|R_{AH,2}|$ at B = 0.1 T.

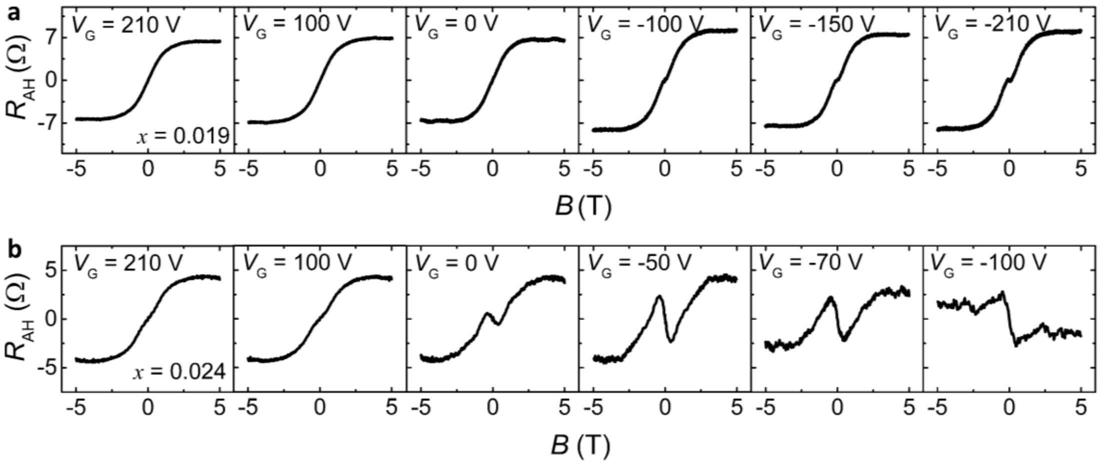

Supplementary Figure 4. Gate-voltage tuning of the anomalous Hall effect in two lightly doped ($Bi_{1-x}Mn_x)_2Se_3$ thin films. (a) AH resistances of Sample H (x = 0.019) at various gate voltages. A small kink structure begins to emerge when V_G is decreased to -100 V, and becomes slightly more pronounced at $V_G = -210$ V. The sheet electron densities determined from the Hall effect data are $n_s = 0.64\times10^{13}$ cm^{-2} and 0.53×10^{13} cm^{-2} at $V_G = -100$ V and -210 V, respectively. (b) AH resistances of Sample D (x = 0.024). At $V_G = 0$, the kink structure is already clear, and the corresponding sheet electron density is $n_s = 0.48\times10^{13}$ cm^{-2}. At large negative gate voltages, the magnitude of the negative R_{AH} component becomes larger than that of the positive one. Both sets of the data are qualitatively same as other lightly doped samples, for instance, Sample C shown in Figure 3 in the main text.

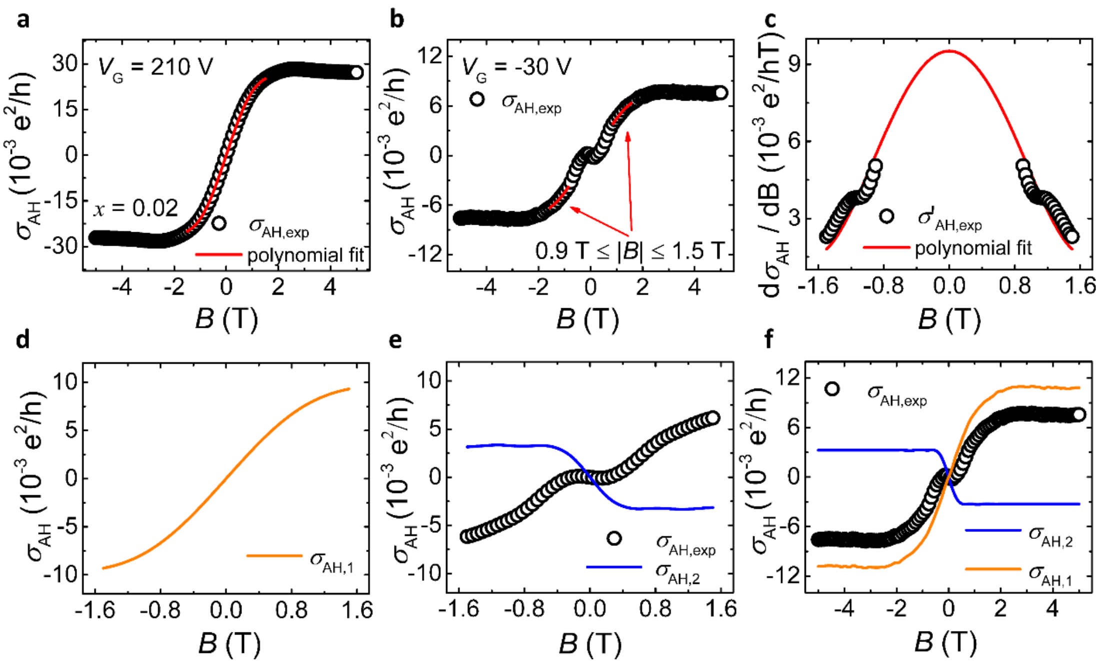

Supplementary Figure 5. Illustration of the first method for separating the two anomalous Hall components with the data of Sample C (x = 0.02). (a) The AH conductivity at V_G = 210 V (black open circles) and a polynomial fit (red line) to the fifth order, i.e. $\sigma_{AH}(B) = a(B + bB^3 + cB^5)$. It is dominated by the positive component, $\sigma_{AH,1}$. (b) The AH conductivity at V_G = –30 V (open circles), in which the positive and negative components coexist. The segments (0.9 < |B| < 1.5 T) used for the polynomial fit shown in panel (c) are marked with red lines. (c) Derivative of the total AH conductivity and the best fit to $\sigma'_{AH}(B) = a(B + 3bB^2 + 5cB^4)$. Only a is the fitting parameter. (d) Calculated $\sigma_{AH,1}$ values by using parameters a, b and c extracted from the above fits. (e) Negative component $\sigma_{AH,2}(B)$ (blue line) obtained by subtracting $\sigma_{AH,1}$ from the total AH conductivity $\sigma_{AH,exp}$ (open circles). (f) The positive component (orange line) in the full range of magnetic fields, obtained with $\sigma_{AH,1}(B) = \sigma_{AH,exp}(B) - \sigma_{AH,2}(B)$.

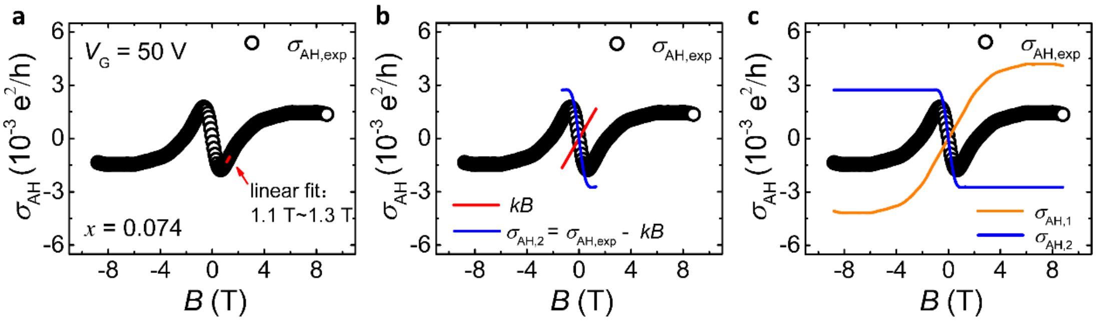

Supplementary Figure 6. Illustration of the second method for separating the two anomalous Hall components with the data of Sample F (x = 0.074). (a) Magnetic field dependence of AH conductivity at V_G = 50 V (open circles). A linear fit is carried out in the field range of B = 1.1 - 1.3 T, in which the negative component $\sigma_{\mathrm{AH,2}}(B)$ is nearly constant due to the low saturation field of the surface magnetization. A slope k = $\mathrm{d}\sigma_{\mathrm{AH}}/\mathrm{d}B$, related to the variation of bulk magnetization with the magnetic field, can be determined from the fit. The negative AH component $\sigma_{\mathrm{AH,2}}(B)$ (blue line) obtained by subtracting $\sigma_{\mathrm{AH,1}}(B) \approx kB$ (red line) from the total AH conductivity $\sigma_{\mathrm{AH,exp}}(B)$ (open circles) at $|B| < 1.3$ T. The linear approximation can be justified by the magnetic field dependence of the AH conductivity that is dominated by the positive component $\sigma_{\mathrm{AH,1}}$ (see, for instance, Figure 3 and Supplementary Figure 4), thanks to the high saturation field ($B_s > 2$ T) in perpendicular magnetic fields due to the in-plane anisotropy of the bulk magnetization. (c) The positive AH component in the full range of magnetic fields (orange line), obtained with $\sigma_{\mathrm{AH,1}}(B) = \sigma_{\mathrm{AH,exp}}(B) - \sigma_{\mathrm{AH,2}}(B)$.

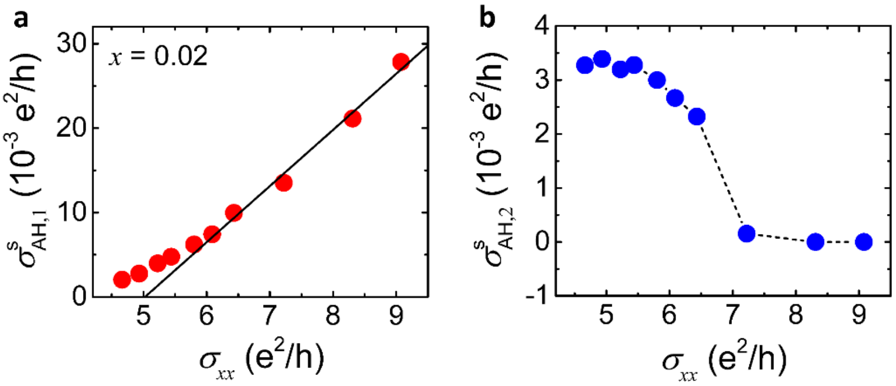

Supplementary Figure 7. Validity of the second method for separating the two anomalous Hall components. Panels (a) and (b) display the magnitudes of the saturated AH conductivities, $\sigma^{\mathrm{s}}_{\mathrm{AH,1}}$ and $\sigma^{\mathrm{s}}_{\mathrm{AH,2}}$, extracted for Sample C (x = 0.02). Both the positive (panel a) and the negative (panel b) AH components, plotted as a function of longitudinal conductivity, σ_{xx} , are consistent with those obtained with the first method (Figure 5b, c in the main text).

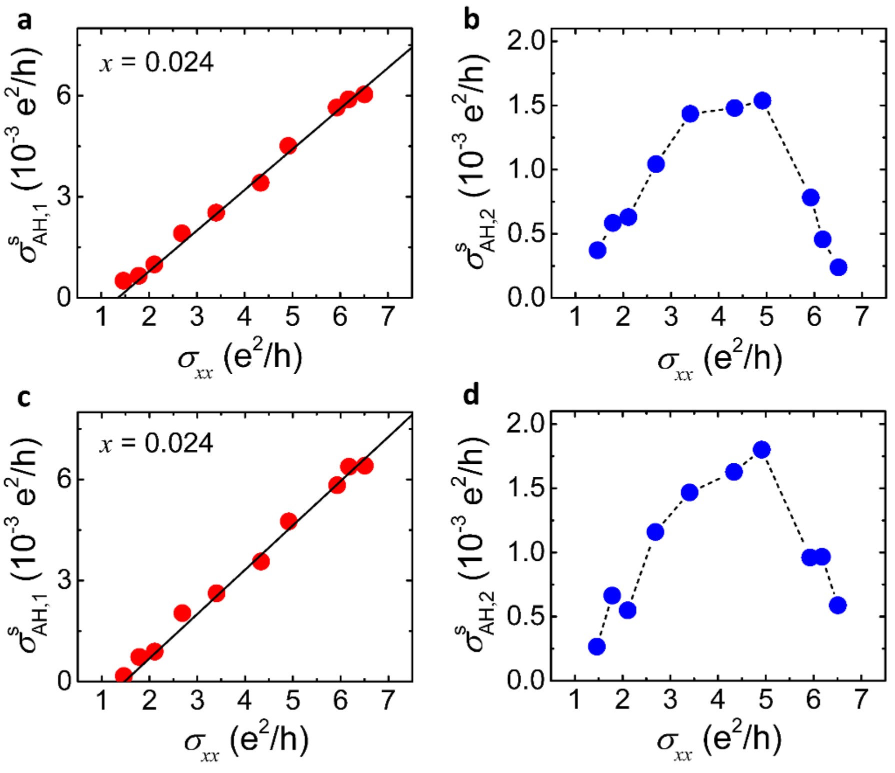

Supplementary Figure 8. Equivalence of the two methods for separating the two AH components of Sample D (x = 0.024). (a, b) The $\sigma^{\mathrm{s}}_{\mathrm{AH,1}}$ and $\sigma^{\mathrm{s}}_{\mathrm{AH,2}}$ values obtained with the first method (see Supplementary Figure 5 and Supplementary Note 2). (c, d) The σ_{xx} dependences of $\sigma^{\mathrm{s}}_{\mathrm{AH,1}}$ and $\sigma^{\mathrm{s}}_{\mathrm{AH,2}}$ extracted with the second method (see Supplementary Figure 6). The negative component has a maximum at $\sigma_{xx} \sim 5e^2/h$, consistent with the trend jointly displayed by the data of Samples C and F (Figures 5 & 6 in the main text).

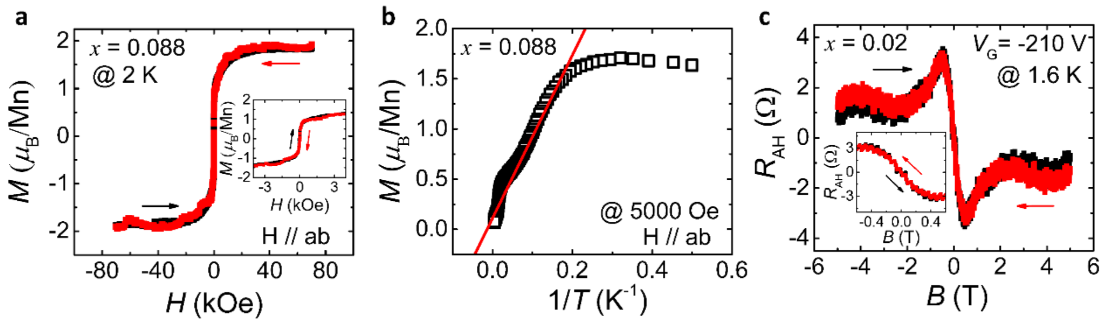

Supplementary Figure 9. Magnetic properties of a $(Bi_{1-x}Mn_x)_2Se_3$ thin film with x = 0.088. (a) Magnetization of a 70 nm thick $(Bi_{1-x}Mn_x)_2Se_3$ film with x = 0.088 measured at T = 2 K in the in-plane magnetic fields. The inset shows that no hysteresis can be clearly resolved within the resolution of the superconducting magnet. (b) Magnetization plotted as a function of $1/T$. (c) Anomalous Hall resistances of Sample C (x = 0.02) record at V_G = –210 V and T = 1.6 K in perpendicular magnetic fields. No clear hysteresis can be resolved either.

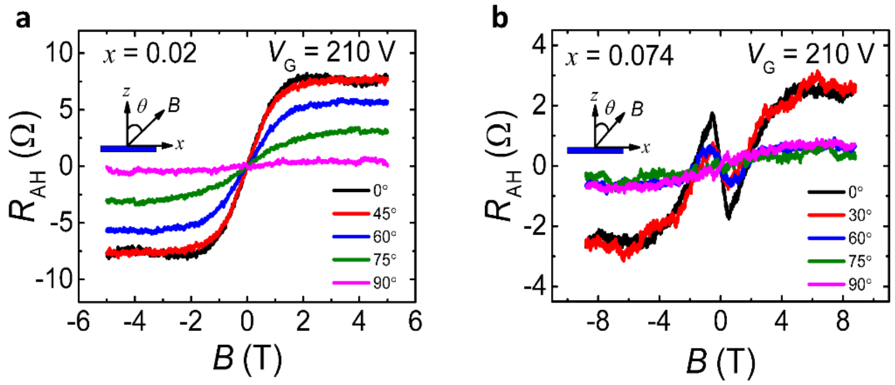

Supplementary Figure 10. Anomalous Hall resistances of two $(Bi_{1-x}Mn_x)_2Se_3$ samples in tilted magnetic fields. (a) R_{AH} of Sample C (x = 0.02). Only when the tilt angle θ is greater than 45°, the magnitude of R_{AH} decreases substantially with increasing θ. (b) Magnetic field dependences of R_{AH} for Sample F (x = 0.074). When the magnetic field is in the out-of-plane direction (θ = 0°), the positive and negative components are comparable in magnitude. Both components respond to the field tilting sensitively, but they exhibit different dependences on θ. For instance, the magnitude of the negative component drops more quickly with increasing θ than the positive component, when the tilt angle is not large. The data in both panels (a) and (b) were taken at T = 1.6 K at V_G = 210 V. The tilt angle θ is defined as the angle between the magnetic field and the normal of the film plane.

Supplementary Note 1: Nonlinear Hall effects in $(Bi,Mn)_2Se_3$ thin films

The Hall resistance in a magnetic material comprises two terms, namely $R_{yx}(B) = R_O(B)+R_{AH}(M)$, where $R_O(B) = R_H B$ is the ordinary Hall resistance, and $R_{AH}(B) = R_s M(B)$ is the anomalous Hall (AH) resistance, which is proportional to the sample magnetization M. Strictly speaking, neither $R_O(B)$ nor $R_{AH}(B)$ is linear in the magnetically doped topological insulator (TI) thin films. The AH resistances in Mn-doped Bi_2Se_3 thin films are usually much smaller than the ordinary Hall resistances, which sometimes contain a nonlinear component comparable to $R_{AH}(B)$ in magnitude. Therefore, a lot of care must be taken when separating the AH component from the measured Hall resistances.

Supplementary Figure 1 shows the Hall resistances of a typical Bi_2Se_3 thin film in magnetic fields up to 15 T. The low field data can be fitted quite well to a linear law. When B > 5 T, the Hall resistance begins to deviate from the linear form clearly. Such a nonlinear Hall effect can be attributed to the coexistence of multiple types of carriers in the sample, namely the electrons on both top and bottom surfaces and in the bulk. This can be understood straightforwardly with the semiclassical two band model, in which the Hall resistance can be written as

$$R_{yx} = \frac{B}{e}\frac{(n_1\mu_1^2+n_2\mu_2^2)+(n_1+n_2)\mu_1^2\mu_2^2B^2}{(|n_1|\mu_1+|n_2|\mu_2)^2+(n_1+n_2)^2\mu_1^2\mu_2^2B^2}, \quad (1)$$

where n_i and μ_i $(i = 1,2)$ are the density and mobility of the ith type of carriers, respectively. When the magnetic field is sufficiently small, i.e. $\mu_1 B \ll 1$ and $\mu_2 B \ll 1$, the B^2 terms in Supplementary Equation (1) can be dropped, and the Hall resistance varies linearly with B. The deviation from the linear dependence in higher magnetic fields (typically $\mu B \sim 0.1$ or larger, with $\mu = \max(\mu_1, \mu_2)$) can be attributed to non-negligible B^2 terms in the above equation.

In undoped or lightly Mn-doped Bi_2Se_3 thin films (e.g. Samples A-D), the carrier mobilities are on the order of 10^2 $cm^2/V{\cdot}s$ (See Table 1 in the main text), and the ordinary Hall effect (OHE) has good linearity in magnetic fields up to B ~ 5 T. In more

heavily doped $(Bi,Mn)_2Se_3$ thin films (e.g. Samples F & G), the carrier mobilities are much lower (See Table 1 in the main text), and consequently the OHE can remain linear for a much wider range of magnetic fields (up to ~10 T or higher). This provides room to select a magnetic field range below the upper limit for the linear OHE, but high enough to make the low temperature magnetization to saturate or become weakly dependent on the magnetic field. A linear fit of $R_{yx}(B)$ in this range of magnetic fields thus provides a convenient means to obtain a reasonable approximation to the ordinary Hall resistance, i.e. $R_{\mathrm{O}}(B) \approx R_{\mathrm{H}}B$, where R_{H} takes the slope value determined from the linear fit. It follows that the AH resistance can be obtained with $R_{\mathrm{AH}}(B) \simeq R_{yx}(B) - R_{\mathrm{H}}B$. In this work, a magnetic field range of 5-9 T was used for extracting R_{H} in the samples with high doping levels (e.g. Sample F, $x = 0.074$). In contrast, the field range is lowered to 3-5 T for the lightly doped samples (e.g. Sample C, $x = 0.02$) because of the nonlinear part of OHE is no longer negligible for the magnetic fields above 5 T.

Another method of extracting the AH resistance is to utilize different temperature dependences of the magnetization and OHE. Supplementary Figure 2a shows that the nonlinear part of Hall resistance, R_{NL}, is nearly independent of the temperature for an undoped Bi_2Se_3 thin film (Sample A). In contrast, for a lightly doped $(Bi,Mn)_2Se_3$ thin film ($x = 0.018$, Sample B), R_{NL} is strongly dependent on temperature at T < 25 K, but becomes temperature independent at $T \geq 25$ K, as shown in Supplementary Figure 2b. It is remarkable that the R_{NL} curves for $T = 25$, 50, 100 and 150 K fall onto each other nearly perfectly. This suggest that in this wide temperature range the magnetization (presumably in the paramagnetic phase) is negligible, and the nonlinear part of OHE is independent of temperature. Therefore, one could simply use a high temperature (25-150 K) $R_{\mathrm{NL}}(B)$ curve as the OHE background to extract the AH resistances at lower temperatures, namely $R_{\mathrm{AH}}(B) = R_{\mathrm{NL}}(B,T) - R_{\mathrm{NL}}(B)|_{T=150\ \mathrm{K}}$. The result for Sample B is depicted in Supplementary Figure 2c, which shows that the R_{AH} reaches saturation in a magnetic field of ~ 5 T at $T = 1.6$ K. A linear fit of the raw Hall effect curve above this field would be ideal to remove the OHE component from the total Hall resistance signal,

if the OHE could remain linear. Unfortunately, this is not case for the lightly doped samples due to the relatively high electron mobilities (e.g. $\mu \approx 327$ cm^2/V·s for Sample B, see Table 1 in the main text). A good comprise is to use a field range of 3-5 T, in which the OHE remains linear and the magnetization is close to saturation so that the small curvature in $R_{AH}(B)$ does not introduce significant error. This method, which was used to extract the $R_{AH}(B)$ data shown in Figures 2-3 in the main text, is more convenient than making multiple measurements at different temperatures. Moreover, when a back-gate voltage is applied, additional complications arise due to the temperature dependent dielectric constant of the $SrTiO_3$ substrate. Strictly speaking, using the temperature dependence to extract the AH resistances is limited to zero gate voltage. For the lightly doped $(Bi,Mn)_2Se_3$ samples, the method relying on the linear fit slightly below 5 T offers an effective means to extract the AH resistances at various gate voltages (e.g. Figure 3 in the main text). Nevertheless, the temperature dependent data taken at $V_G = 0$ (e.g. Supplementary Figure 2) can be used to check validity of the first method of subtracting the OHE (i.e. the linear approximation approach). Indeed, these two methods give consistent results on the extracted AH resistances.

Supplementary Note 2: Separation of two anomalous Hall components

In the following, we describe two methods used for separating the two AH components with opposite signs. The first method can be used to analyze the data of lightly doped (Bi,Mn)$_2$Se$_3$ thin films, in which the AH resistance is dominated by the positive component when the electron density is sufficient high. The second method utilizes the large difference in the saturation fields of the two components and can be used for samples with all Mn-doping levels. Here, the total AH conductivity is written as $\sigma_{AH}(B) = \sigma_{AH,1}(B) + \sigma_{AH,2}(B)$, in which $\sigma_{AH,1}(B)$ and $\sigma_{AH,2}(B)$ denote the positive and negative components, respectively.

The first method is illustrated in Supplementary Figure 5 with the data of Sample C ($x = 0.02$). The $\sigma_{AH}(B)$ data at $V_G = +210$ V only has the positive component (panel a). An approximate analytical function for $\sigma_{AH,1}(B)$ can be obtained with a fit to the following polynomial: $\sigma_{AH}(B) = a(B + bB^3 + cB^5)$. Its derivative is $\sigma'_{AH}(B) = a(1 + 3bB^2 + 5cB^4)$. For the gate voltages exhibiting two-component AH effect, for instance, $V_G = -30$ V shown in panel b, the total AH conductivity needs to be separated into a positive component with higher saturation field ($B_{s,1}$) and a negative component with lower saturation field ($B_{s,2}$). For magnetic fields in between, i.e. $B_{s,2} < B < B_{s,1}$, $\sigma'_{AH}(B)$ is solely contributed by $\sigma_{AH,1}(B)$. Panel c shows the fit of $\sigma'_{AH}(B)$ data in a range of 0.9-1.5 T to the derivative function $\sigma'_{AH}(B) = a(1 + 3bB^2 + 5cB^4)$. In the fit, coefficients b and c are fixed to the values determined from the polynomial fit depicted in panel a, so the coefficient a is the only fitting parameter. The positive AH component at $V_G = -30$ V is then calculated with $\sigma_{AH,1}(B) = a(B + bB^3 + cB^5)$ (panel d). It follows that the negative component can be obtained by subtracting the positive component from the total AH conductivity: namely $\sigma_{AH,2}(B) = \sigma_{AH}(B) - \sigma_{AH,1}(B)$ (panel e). The extracted AH components in the full field range are plotted in panel f. It shows that $\sigma_{AH,2}$ saturate at $B \approx 0.5$ T, thus justifying the magnetic field range used for the derivative fit.

The second method for disentangling the two AH components is shown in Supplementary Figure 6 with data taken from Sample F ($x = 0.074$) at $V_G = 50$ V. In

such heavily doped samples, the first method cannot be applied because the negative component $\sigma_{AH,2}(B)$ is substantial even at a high positive gate voltage (panel a). The second method is based on the fact that the saturation field of negative component is considerably lower than that of the positive component, i.e. $B_{s,2} < B_{s,1}$. The derivative of the total AH conductivity, $d\sigma_{AH}/dB$, is hence solely contributed by the positive component $\sigma_{AH,1}(B)$ for the fields $B_{s,2} < B < B_{s,1}$. As shown in panel b, if the magnetic field is not too high (e.g. $|B| < 1.3$ T), a linear approximation can be taken for $\sigma_{AH,1}(B)$, namely $\sigma_{AH,1} \approx kB$, where $k = d\sigma_{AH}/dB$ can be determined from a linear fit of the total AH conductivity for the fields $1.1 < B < 1.3$ T. With the low field $\sigma_{AH,1}(B)$ determined approximately, the negative component can now be obtained by doing the following subtraction: $\sigma_{AH,2}(B) = \sigma_{AH}(B) - kB$. In panel c, we use the average value of $\sigma_{AH,2}(B =$ 1.1-1.3 T) shown in panel b as the saturated AH conductivity to extend the $\sigma_{AH,2}(B)$ curve to higher magnetic fields. The $\sigma_{AH,1}(B)$ values can then be obtained by a similar subtraction for the full range of magnetic fields.

Both methods have been applied to Sample C ($x = 0.02$). As shown in Supplementary Figure 7, the result obtained with the second method produces the results in good agreement with those acquired with first method (See Figure 5 in the main text). Supplementary Figure 8 further shows that consistent results can also be obtained with these two methods for Sample D ($x = 0.024$).

Supplementary Note 3: Magnetic order in $(Bi_{1-x}Mn_x)_2Se_3$ thin films

Previous bulk sensitive measurements[1-4], such as SQUID magnetometry and ferromagnetic resonance, have established that there is a weak ferromagnetic order with an in-plane easy magnetization axis in the ungated $(Bi,Mn)_2Se_3$ thin films. Our in-magnetization measurements (Figure 1c in the main text) are consistent with those results. In the lightly doped $(Bi,Mn)_2Se_3$ thin films, the AHE is dominated by the positive component arising from the bulk magnetization. The AH resistance therefore provides a sensitive probe to the bulk magnetization in the perpendicular magnetic fields. As shown in Supplementary Figure 2c, both the magnetic field and the temperature dependences of the AH resistance agree with the magnetization measurements reported in Ref. 4, in which a ferromagnetic transition temperature T_C of 5.5 K was determined by an Arrott plot analysis. Supplementary Figure 2d shows that the low field slope of the AH resistance, dR_{AH}/dB, which is presumably proportional to the magnetic susceptibility, has a linear dependence on $1/T$ at $T > 5$ K. The deviation from such a paramagnetic behavior is consistent with the previous conclusion of the ferromagnetic order at low temperatures[4]. Similar phenomenon has also been observed in the temperature dependence of the in-plane magnetization in a fixed magnetic field, as shown in Supplementary Figure 9b.

As to the surface magnetism, the only experimental technique that has succeeded so far in detecting magnetic signals from the surface of the $(Bi,Mn)_2Se_3$ thin films is the X-ray magnetic circular dichroism (XMCD). It was reported in Ref. 4 that the XMCD measurements, which probe the out-of-plane magnetization near the surface, provide evidence for ferromagnetic order with a T_C of ~10 K for a $(Bi_{1-x}Mn_x)_2Se_3$ thin film with $x = 0.04$ and 0.08. Such measurements were, however, limited to the temperatures above 5 K and lack of detailed magnetic field dependences. The negative AH components observed in our work thus provide valuable information on the surface magnetism. For instance, Supplementary Figure 3a shows detailed measurements of the AHE in Sample F ($x = 0.074$) at in a wide range of temperatures and up to very high magnetic fields. The AH resistance contains a substantial negative component, whose

magnitude decreases rapidly with increasing temperature (see Supplementary Figures 3b-d). From the temperature dependence of low field R_{AH}, a transition temperature of ~15 K can be obtained.

An important aspect of this work is the observation of the two-component AHE, which suggests that the surface and bulk magnetic orders could coexist in magnetically doped TIs. It is generally accepted that the TI surface would favor an out-of-plane magnetization[5-7]. The lower saturation field of the negative AH component than that of the positive component seems to be consistent with this. Our experiment also supports an in-plane easy magnetization axis for the bulk states, in agreement with previous works[1-4]. However, it would be oversimplified to treat the magnetic structure in $(Bi,Mn)_2Se_3$ thin films as a sum of independent surface and bulk magnetizations with out-of-plane and in-plane anisotropies, respectively. After all, the thickness of the films studied this work is only 10 nm. It is reasonable that the spin interactions between the surface and bulk states could lead to more complex spin structures, such as spin canting and non-colinear spin textures. The AHE measurements in tilted magnetic fields suggest that the dependences of both the positive and negative components on the tilt angle cannot be explained as a simple magnetic thin film with either out-of-plane or in-plane anisotropy. As shown in Supplementary Figure 10a, the positive AH component in a lightly doped sample (x = 0.02, Sample C) changes very little when the magnetic field is tilted 45° from the perpendicular orientation. Whereas for a heavily doped sample (x = 0.074, Sample F), a 30° field tilt causes very large change in the negative AH component. It is also interesting to note that the negative AH component does not vanish completely, and even reverses sign when the magnetic field is aligned parallel to the thin film plane. These results might be signatures for spin canting or other complex spin structures originating from the competition between surface and bulk magnetic orders. More theoretical and experimental efforts are hence necessary to unveil the rich spin phenomena in the magnetically doped TIs.

References:

1. Zhang, D. M. *et al.* Interplay between ferromagnetism, surface states, and quantum corrections in a magnetically doped topological insulator. *Phys. Rev. B* **86**, 205127 (2012).
2. von Bardeleben, H. J. *et al.* Ferromagnetism in Bi_2Se_3: Mn epitaxial layers. *Phys. Rev. B* **88**, 075149 (2013).
3. Collins-McIntyre, L. J. *et al.* X-ray magnetic spectroscopy of MBE-grown Mn-doped Bi_2Se_3 thin films. *AIP Advances* **4**, 127136 (2014).
4. Sánchez-Barriga, J. *et al.* Nonmagnetic band gap at the Dirac point of the magnetic topological insulator $(Bi_{1-x}Mn_x)_2Se_3$. *Nat. commun.* **7**, 10559 (2016).
5. Rosenberg, G. & Franz, M. Surface magnetic ordering in topological insulators with bulk magnetic dopants. *Phys. Rev. B* **85**, 195119 (2012).
6. Liu, Q., Liu, C. X., Xu, C., Qi, X. L. & Zhang, S.-C. Magnetic impurities on the surface of a topological insulator. *Phys. Rev. Lett.* **102**, 156603 (2009).
7. Efimkin, D. K. & Galitski, V. Self-consistent theory of ferromagnetism on the surface of a topological insulator. *Phys. Rev. B* **89**, 115431 (2014).
